# Aescin-Cholesterol Complexes in DMPC Model Membranes: A DSC and Temperature-Dependent Scattering Study

**DOI:** 10.1038/s41598-019-41865-z

**Published:** 2019-04-03

**Authors:** Ramsia Sreij, Carina Dargel, Ralf Schweins, Sylvain Prévost, Rajeev Dattani, Thomas Hellweg

**Affiliations:** 1 Physical and Biophysical Chemistry, Bielefeld University, Universitätsstr. 25, D-33615 Bielefeld, Germany; 2 Institut Laue-Langevin, DS/LSS, 71 avenue des Martyrs, 38042 Grenoble Cedex 9, France; 3 ESRF-The European Synchrotron, 71 Avenue des Martyrs, 38043 Grenoble Cedex 9, France

## Abstract

The saponin aescin, a mixture of triterpenoid saponins, is obtained from the seeds of the horse chestnut tree *Aesculus hippocastanum*. The *β*-form employed in this study is haemolytically active. The haemolytic activity results from the ability of aescin to form strong complexes with cholesterol in the red blood cell membrane. In this study, we provide a structural analysis on the complex formation of aescin and cholesterol when embedded in a phospholipid model membrane formed by 1,2-dimyristoyl-*sn*-glycero-3-phosphocholine (DMPC). In this work, the temperatures investigated extend from DMPC’s L_*β*′_ to its L_*α*_ phase in dependence of different amounts of the saponin (0–6 mol% for calorimetric and 0–1 mol% for structural analyses) and the steroid (1–10 mol%). At these aescin contents model membranes are conserved in the form of small unilamellar vesicles (SUVs) and major overall structural modifications are avoided. Additionally, interactions between aescin and cholesterol can be studied for both phase states of the lipid, the gel and the fluid state. From calorimetric experiments by differential scanning calorimetry (DSC), it could be shown that both, the steroid and the saponin content, have a significant impact on the cooperative phase transition behaviour of the DMPC molecules. In addition, it becomes clearly visible that the entire phase behaviour is dominated by phase separation which indeed also depends on the complexes formed between aescin and cholesterol. We show by various methods that the addition of cholesterol alters the impact of aescin on structural parameters ranging from the acyl chain correlation to vesicle-vesicle interactions. While the specific saponin-phospholipid interaction is reduced, addition of cholesterol leads to deformation of SUVs. The analyses of the structures formed were performed by wide-angle X-ray scattering (WAXS), small-angle X-ray scattering (SAXS), and small-angle neutron scattering (SANS).

## Introduction

**Figure 1:**
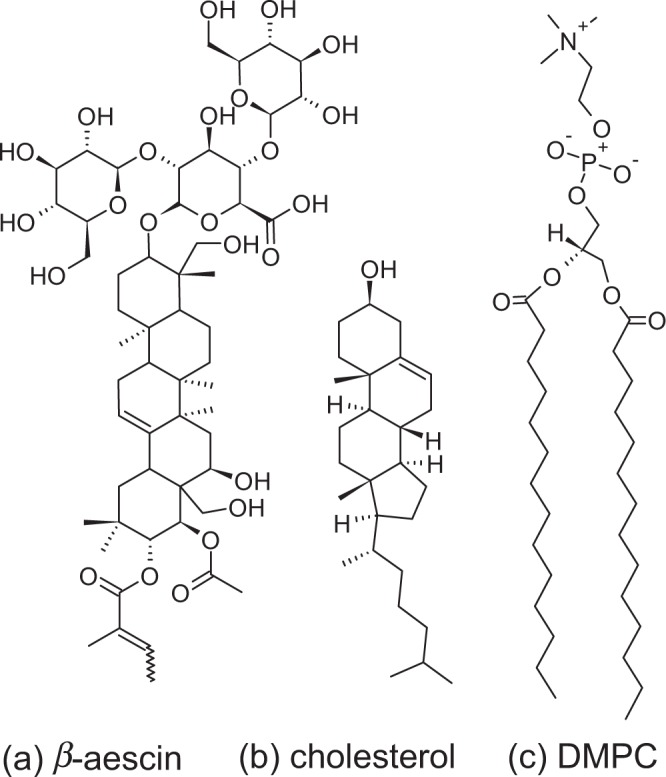
Schemes showing the molecular structures of (**a**) *β*-aescin, (**b**) cholesterol, and (**c**) DMPC.

A mixture of saponins can be isolated from the seeds of the horse chestnut tree *Aesculus hippocastanum*^1,2^. From this mixture, two crystalline products are saparable: *aescin* (haemolytic) and *prosapogenin* (non-haemolytic)^1,3^. Aescin is a mixture of triterpenoid saponins^4^. It exists in two forms, the *α-* and the *β*-form from which the latter is haemolytically active and the compound of interest in this study^1^. The aescin molecule is constituted of a large and well-defined head group made of one glucuronic acid and two glucose molecules linked to a lipophilic sapogenin^1,5,6^. The aglycones of aescin are derivatives of proto-ascigenin, acylated by acetic acid at C-22 and by either angelic or tiglic acids at C-21^1^. They can be distinguished by their melting point, the specific rotation, haemolytic index, and solubility in water^1^. To the triterpenoid backbone, additional polar groups are attached giving this molecule a polar and an apolar side. Figure 1(a) shows the molecular structure of *β*-aescin.

Saponins, such as aescin, are consumed on a daily basis as they are found in common foods, such as peanuts, spinach, tomatoes and tea^7,8^. Aescin possesses anti-inflammatory and anti-oedematous properties^9^. Therefore, it is used to treat chronic venous insufficiency (CVI). The anti-oedematous effect is attributed to aescin, because in case of a hypoxia it i.a. antagonizes the reduction of the adenosine triphosphate (ATP). This counteracts the degradation of the endothelial cells that separate the blood from the surrounding tissue^1,10^. Additionally, in case of CVI treatment, the presence of aescin reduces the permeability of blood capillaries by non-competitively inhibiting the enzyme hyaluronidase. This enzyme is responsible for the degradation of hyaluronic acid, which stabilizes the capillaries^6,11^. Furthermore, the venous tension has been shown to increase because the presence of aescin results in an increased concentration of prostaglandin F2. This hormone is responsible for activating the smooth muscles of the veins^1,12^. A different key feature of some saponins including aescin is the haemolytic activity. It results from the interaction through complexation with cholesterol in the red blood cell membrane^13–18^. The amount of this important steroid can be as high as 50 mol% in biological cell membranes^19^. The molecular structure of cholesterol is shown in Fig. 1(b).

In early reports it has been shown that complexation of saponins and cholesterol is monomolecular and that cholesterol can be used as target for saponins^20,21^. Furthermore, the interaction of saponins with cholesterol has gained much attention not only due to the pharmacological relevance of these two compounds but also due to the complex phase behaviour when these additives are in a phospholipidic environment. The complex phase behaviour of cholesterol-saponin mixtures is subject of earlier works by other groups^22,23^. In these studies, the two saponins digitonin or *Quil A* were intensively investigated^20–23^. In combination with phospholipids, these ternary systems (with *Quil A*) exhibit a broad variety of structures ranging from spherical and worm-like micelles to cage-like structures known as immune stimulating complex matrices (ISCOMs). The formed structures are concentration-dependent. The interaction of especially aescin with cholesterol and phospholipids has gained special interest in pharmacological applications. It has been found that the pharmacological activity is enhanced when aescin and the phospholipid are complexed with cholesterol, as shown by Bombardelli *et al*.^24^ adsorption via an oral route is vastly improved because aescin-cholesterol complexes are more lipophilic than the pure saponins. Also, the haemolytic index is reduced. In their study, the molar ratio of the phospholipid to aescin and cholesterol ranged from 0.5 to 2.

The interaction of the saponin *β*-aescin with cholesterol in 1,2-dimyristoyl-*sn*-glycero-3-phosphocholine (DMPC) model membranes in the form of small unilamellar vesicles (SUVs) is subject of the present work. The molecular structure of DMPC is shown in Fig. 1(c). By using this phospholipid, interactions in both the gel and fluid phases are easily experimentally accessible. Moreover, these results can be directly compared to our previous results^25–27^. The methods we employed were small-angle X-ray scattering (SAXS), small-angle neutron scattering (SANS), wide-angle X-ray scattering (WAXS), and differential scanning calorimetry (DSC). The concentration range, given in respect to the phospholipid content, for cholesterol and aescin was 1–10 mol% and 0–6 mol%, respectively. By using these relatively low cholesterol contents the aescin-cholesterol interactions can be investigated at different phase states of DMPC (L_*β*′_ and L_*α*_) before the phase states are strongly altered by the steroid^28–30^. Phase diagrams of phosphatidylcholine bilayers with different amounts of cholesterol do not show any impact on the phase transition temperature for steroid contents below 1 mol%. Therefore, at very low cholesterol content (i.e. 1 mol%) the distribution of the steroid can be considered as rather homogeneous in the bilayer^28–30^. The structural analyses were restricted to aescin contents <1 mol% to avoid major structural modifications such as decomposition of the membrane induced by the saponin^25,26^. Hence, at these conditions the aescin-cholesterol interactions are studied while the vesicular character of the samples is still maintained. Structures such as ICOMs require much higher amounts of steroid and saponin^22,23,31^.

Using DSC we confirm that there is a strong interaction between saponin and phospholipid and its complexation with cholesterol and show how the cooperative phase transition is altered depending on saponin and steroid content. Structural analysis was done on the sub-nm scale by WAXS. Here, there is a clear impact on the acyl chain correlation distance (*d*_*WAXS*_) and the area per lipid molecule (*A*_*L*_). These are outlined as function of the main phase transition temperature *T*_*m*_ and sample composition. SANS and SAXS provide information on larger length scale and confirm binding of cholesterol to aescin and severe impact on vesicle-vesicle interactions. Cholesterol enhances aggregation and leads to structural deformation of SUVs.

## Results

### Differential scanning calorimetry (DSC)

**Figure 2:**
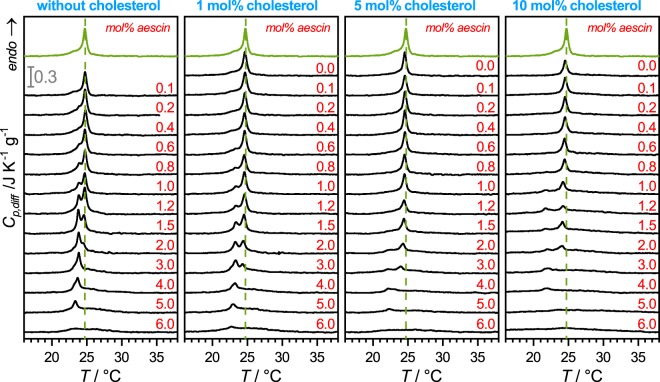
DSC data: Endotherms for (**a**) the binary system DMPC/aescin and the ternary systems containing (**b**) 1 mol%, (**c**) 5 mol%, and (**d**) 10 mol% of additional cholesterol. The aescin content was varied for all samples between 0 mol% and 6 mol%, as indicated by the red numbers. The dashed green line indicates the peak position of pure DMPC SUVs (green endotherm). The scale bar applies for all panels. Experiments were performed from 10–40 °C. Endotherms are zoomed for better visibility of the peaks.

DSC experiments were performed between 10 °C and 40 °C to investigate the temperature-induced and heat-flow associated conformational changes of the aliphatic chains of the phospholipid (*all-trans* to *partially gauche* configuration). In particular, DMPC undergoes the main phase transition at 23.6 °C^25^ at which the aliphatic chains transform form the ripple P_*β*′_ phase to the fluid L_*α*_ phase. The rigid gel L_*β*′_ phase is reached at 10 °C^32^. In this experiment, especially the impact of added molecules influencing particularly this transition becomes clear. The aescin contents are varied between 0 mol% and 6 mol% with respect to the phospholipid content. The steroid content was maintained constant for each data set at 0 mol%, 1 mol%, 5 mol%, and 10 mol%. The endotherms obtained are presented in Fig. 2(a–d), respectively.

The green curve in all panels corresponds to pure DMPC SUVs. The dashed green line indicates the peak position of the peak maximum of these SUVs. Figure 2(a) shows the thermograms obtained for the binary system DMPC/aescin. A detailed analysis of this system has already been discussed in previous works^25,26^. However, as already elucidated^25,26^, the main characteristic and the most crucial feature in the aescin-containing system is the upcoming peak at lower temperature with increasing aescin content. The presence of this peak indicates aescin-induced phase separation into saponin-rich and saponin-poor domains within the bilayer. Hence, the different signals detected by DSC correspond to DMPC molecules located in different chemical environments (i.e. aescin-poor and aescin-rich environment). Aescins tendency to phase separate was explained by its molecular structure and its partial structural similarity to bile salt molecules which show similar behaviour^33–38^. Aescin possesses a very large glyconic head group typical for saponins and which was shown for the saponin *Quil A* to strongly interact with the phospholipid headgroups^39^. Additionally, its triterpenic backbone has a polar and an apolar side. When embedded into a rather hydrophobic phospholipid environment, the unilaterally located polar groups are most likely protected from the hydrophobic milieu by turning towards each other. Additionally, molecular dynamics (MD) simulations on the self-assembly of aescin molecules at the water-air interface by Tsibranska *et al*.^40^ revealed that these molecules assemble into compact clusters through short range hydrogen bonds between the sugar moieties, intermediate dipole-dipole, and long-range attractive interactions between hydrophobic aglycones.

While addition of cholesterol alone has no significant influence on the endotherms, the combined presence of cholesterol and aescin has a significant effect, even at 1 mol% cholesterol. This effect is most prominent between 1 mol% and 3 mol% aescin where the effect of a very distinct phase separation is observable. The formation of aescin/cholesterol complexes reduces the amount of free aescin molecules available for interactions with the phospholipid membrane, thereby slowing down the increase of the low-temperature peak on the left-hand side of the main DMPC peak. Additionally, slight signal broadening occurs. Formation of strong saponin/cholesterol complexes was already discussed by other groups and is in good agreement with our findings for the saponin aescin at the cholesterol contents presented^21–24^. Addition of more steroid smears the well-defined phase separation by the aescin and broadens the signal immensely (see same aescin contents in panels (b–d) in Fig. 2). Similar signal broadening was already observed in binary cholesterol/phospholipid systems^19,28^. In these systems the relatively stiff steroid backbone interacts with the lipid membrane by van der Waals interactions so that the natural transition of the hydrocarbon chains is altered. These interactions result in a widened endothermic signal which expands to both, lower and higher temperatures. Another interesting feature is the shift of this peak to much lower temperatures with increasing aescin content, as will be discussed more quantitatively below.

**Figure 3:**
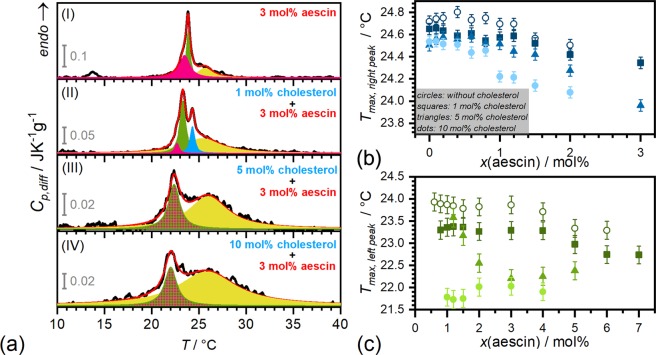
DSC results. (**a**) DSC endotherms of samples with aescin contents of 3 mol%. The cholesterol content is varied between (I) 0 mol%, (II) 1 mol%, (III) 5 mol%, and (IV) 10 mol%. The *blue* signal results from the phase transition of DMPC molecules in a saponin-poor environment, the *green* signal to one in aescin-rich environment. The *pink* signal arises at relatively high aescin contents. The *green* peak with the *pink* pattern is mixture of the single colour signals in panels (I) and (II). The *yellow* signal denotes the signal broadening by cholesterol addition. The red line is the sum of all peaks. Peak maxima obtained from Lorentzian fits for samples without (◦) and with 1 mol% (■), 5 mol% (▲), and 10 mol% (●) cholesterol. The figure depicts the fit results from the *blue*-coloured peak in panel (b) and those of the *green* coloured peak in panel (c), according to the colour encoding in panel (a). Error bars result from instrument precision^65^.

A closer look on the signals of the endotherms at the intermediate aescin content of 3 mol% reveals that the signal is composed of several underlying peaks. These peaks are highlighted by different colours in Fig. 3(a) in panels (I), (II), (III) and (IV) in the systems containing 0 mol%, 1 mol%, 5 mol%, and 10 mol% cholesterol, respectively. The signal is composed of three distinct peaks at this aescin content without addition of cholesterol (panel (I)). The *blue* signal, which mainly denotes the saponin-poor environment, is only visible for a cholesterol content of 1 mol% (panel (II)). This indicates, that little amounts of cholesterol reduce the effect of aescin on the bilayer. The presence of the blue-coloured peak denotes that the amount of aescin-poor domains is higher in the presence of 1 mol% cholesterol. This effect may result from the rather homogeneous distribution of the steroid in the bilayer at these concentrations. The *green* coloured peak, which was found to increase in intensity with increasing aescin contents, represents DMPC molecules which are already located in an aescin-rich environment. It is visible for the system without cholesterol in panel (I) and with 1 mol% in panel (II) of the same figure. At higher cholesterol contents this signal merges with the one indicated by the *pink* signal. The *pink* peak only gains significant intensity for aescin contents above 3 mol% aescin and indicates that high amounts of aescin disturb the cooperative phase transition of DMPC’s hydrocarbon chains at already lower temperature. Major structural reorganization may have a similar effect due to modifications of the surrounding environment of the phospholipids. When cholesterol is added in higher quantities, these two signals merge into a larger one (*green* peak with *pink* pattern). The significant signal broadening which is most presumably induced by the insertion of stiff molecular parts into the bilayer, is represented by a broad *yellow* peak. This contribution evolves at higher cholesterol contents.

Peak maxima of the *blue* and *green* peaks were analysed to monitor the effect of composition on the shift of *T*_*m*_. The results are summarised in Fig. 3(b,c), respectively. The effect of cholesterol shows in both peak maxima analysed, i.e. *T*_*max*,*right peak*_ and *T*_*max*,*left peak*_. Here, the steroid induces a minor but still visible decrease of *T*_*max*,*right peak*_-values in the saponin-poor environment (*blue*), compared to the effect of aescin (see evolution with increasing aescin content). The impact of cholesterol on *T*_*max*,*left peak*_, i.e. in the aescin-rich environment, is much more significant when more cholesterol-aescin complexes are formed. Here, *T*_*max*,*left peak*_ is lowered by almost two degrees at 10 mol% steroid. These results confirm the impact of larger complexes on the phase transition with cooperative nature of DMPC’s hydrocarbon tails.

### Wide-angle X-ray scattering (WAXS)

WAXS shows the impact on structural parameters on the Å-scale for the highest cholesterol content studied and relatively low amounts of the saponin. At these conditions vesicular structures are maintained and not decomposed into smaller structures by the aescin^27,33^ and the effect of cholesterol is highest on the bilayer and the conditions are closest to those in natural cells. WAXS measures the acyl chain correlation distance *d*_*WAXS*_. This is an important parameter in systems containing correlated structures. It can be calculated from the peak position by Eqn. 1.

1 $${d}_{WAXS}=\frac{2\pi n}{{q}_{peak}}.$$

**Figure 4:**
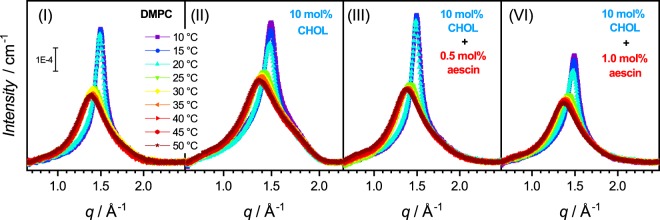
WAXS curves. WAXS spectra of DMPC and aescin and cholesterol containing samples at temperatures between 10 and 50 °C. (I) DMPC and (II–IV) in the presence of 10 mol% cholesterol with 0 mol%, 0.5 mol%, and 1.0 mol% aescin, respectively. The scale bar is valid for all panels.

In this equation, *q*_*peak*_ is the q-value of the Bragg diffraction peak of the *n*th order. Figure 4 shows WAXS diffraction patterns of pure DMPC SUVs in panel (I) and of cholesterol containing systems without and with 0.5 and 1 mol% aescin in panels (II), (III), and (IV), respectively. The temperature range measured is 10 to 50 °C. The effect of the conformation change of the hydrocarbon chains from the L_*β*′_ to the L_*α*_ phase is resolved in temperature steps of 5 °C.

The presence of cholesterol modifies peak shapes and intensities (panels (I) and (II)). The appearance of additional shoulders indicates that the steroid is unevenly distributed in the bilayer by forming domains or rafts on the lateral plane of the membrane, as expected from literature^41^. The addition of as little as 0.5 mol% aescin reduces the effect induced by the cholesterol which confirms the expected complexation of both compounds^24^. Addition of 1 mol% aescin restores the smoother peak broadening with temperature, which is different from the cholesterol-free system shown in Supplementary Fig. S1.

**Figure 5:**
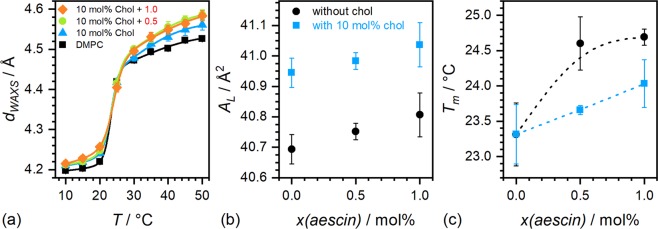
WAXS results of samples with 10 mol% cholesterol: (**a**) Acyl chain correlation distances *d*_*WAXS*_ obtained from peak positions in Fig. 4. The red numbers in the legend are the aescin content in mol% and the solid lines are sigmoidal fits. (**b**) Area per lipid molecule *A*_*L*_ calculated from the position of *q*_*peak*_ of the acyl chain correlation peak at 10 °C by Eqn. 2 while assuming hexagonal packing. (**c**) *T*_*m*_ (WAXS) obtained from the inflection point of the sigmoidal fit in panel (a).

The peak maxima were analysed by Eqn. 1. The results for the cholesterol containing systems are presented in Fig. 5(a) and the ones for the cholesterol-free systems in Supplementary Fig. S2. Different from the cholesterol-free systems (Supplementary Fig. S2) the main impact is not only observed around *T*_*m*_, but *d*_*WAXS*_-values are higher below and above *T*_*m*_. However, at temperatures >30 °C no significant difference between 0.5 mol% and 1 mol% aescin is observable. At temperature <*T*_*m*_ small impact of aescin is visible. In earlier studies we showed that the effect of aescin incorporation into a DMPC membrane is especially visible below *T*_*m*_. The interaction between phospholipid and saponin affects the membrane the most at temperatures where the pure phospholipid would be in the gel state. In its fluid state, the DMPC/cholesterol bilayer is barely modified by the presence of saponin^25,27^. The area per lipid molecule (*A*_*L*_) results from the two-dimensional packing of the phospholipids. At 10 °C, the phospholipids are packed in a hexagonal lattice. Eqn. 2 relates then the position *q*_*peak*_ = *q*_*WAXS*_ of the acyl chain correlation peak with *A*_*L*_^42^.

2 $${A}_{L}=\frac{16{\pi }^{2}}{\sqrt{3}{q}_{WAXS}^{2}}$$

*A*_*L*_ has been calculated for the systems with and without cholesterol. The results are shown as function of aescin content in Fig. 5(b). While the addition of cholesterol increases *A*_*L*_ by around 0.6%, supplementary aescin leads to an increase of 0.25% in both systems. Here, the latter increase occurs within the experimental error bars. Hence, it can be concluded that the effect of cholesterol is much more pronounced and that the presence of complexes formed between aescin and cholesterol increases *A*_*L*_ only slightly. Figure 5(c) shows main phase transition temperatures *T*_*m*(*WAXS*)_ derived from sigmoidal fits (solid lines in Fig. 5(a)) to data points of *d*_*WAXS*_. The transition in cholesterol-free systems occurs at higher temperatures whereby already low amounts as 0.5 mol% have a significant impact on *T*_*m*(*WAXS*)_. This was concluded to result from strong interaction of aescins sugar moieties with DMPC headgroups in former studies^25,27,33^. Addition of only cholesterol has no significant impact on *T*_*m*(*WAXS*)_ whereas additional aescin again leads to a shift of *T*_*m*(*WAXS*)_ to higher temperatures. Compared to the cholesterol-free system, the temperature is shifted by smaller increments hardly detectable within the experimental error bars. This again confirms saponin-steroid interaction in the bilayer.

### Small-angle X-ray scattering (SAXS)

**Figure 6:**
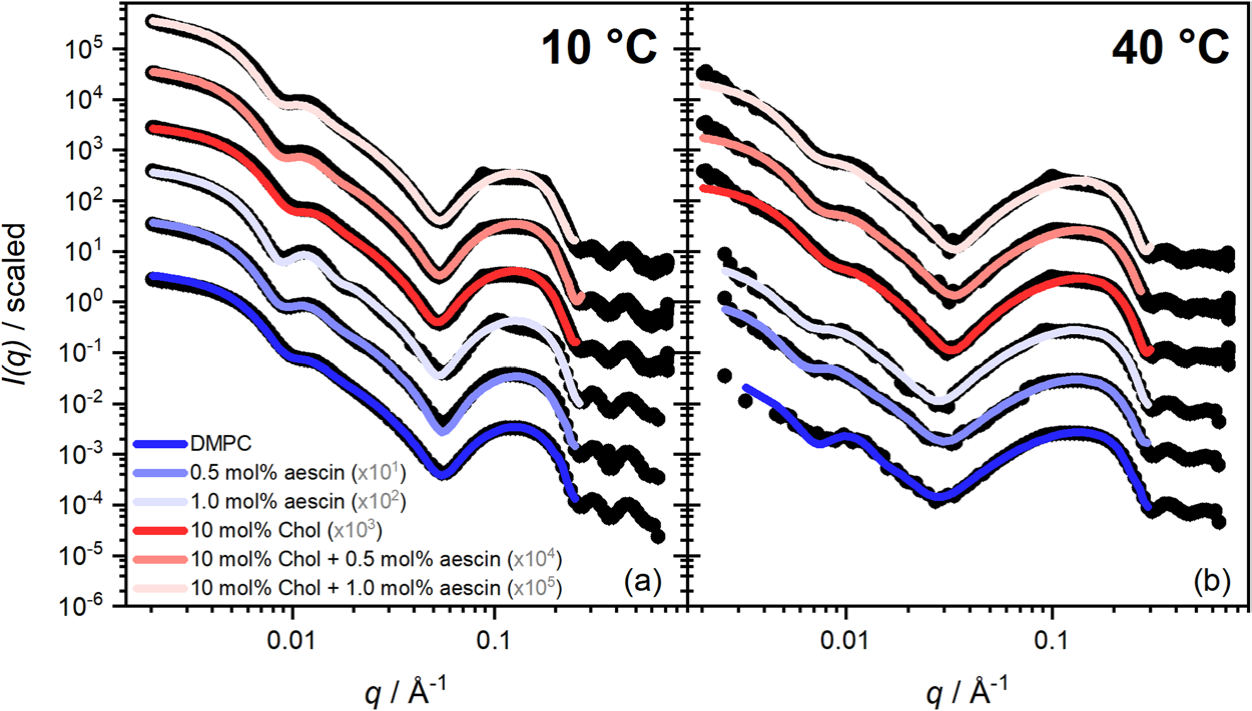
SAXS form factors of DMPC SUVs and SUVs with additives. The cholesterol content is 10 mol%. The aescin content is 0.5 mol% and 1 mol% for samples with and without the steroid at (**a**) 10 °C and (**b**) 40 °C. The SAXS curves for DMPC are on absolute scale (cm^−1^). The other data are scaled by the the factors given in grey. The solid lines are core multi shell (CMS) fits. Additional SAXS curves at temperatures between 10 °C and 50 °C are shown in Supplementary Fig. S4.

The role of cholesterol in the ternary systems studied is outlined by SAXS. Therefore, the same exemplary samples were investigated to scrutinize the thermo-responsiveness of structural parameters from the SAXS perspective. SAXS curves of SUVs yield information about mesoscale structures. In this case, low-*q* (<0.008 Å^−1^) and intermediate-*q* (≈0.01–0.06 Å^−1^) data, yield information about the sample morphology. At larger *q* (0.06–0.3 Å^−1^), information about bilayer parameters and correlated structures is obtained. SAXS curves of samples with 0, 0.5, and 1.0 mol% aescin without and in the presence of 10 mol% cholesterol are shown for temperatures of 10 °C and 40 °C in Fig. 6(a,b), respectively. Additional SAXS curves in the temperature range between 10 °C and 50 °C are shown for same aescin and cholesterol contents in Supplementary Fig. S4.

**Figure 7:**
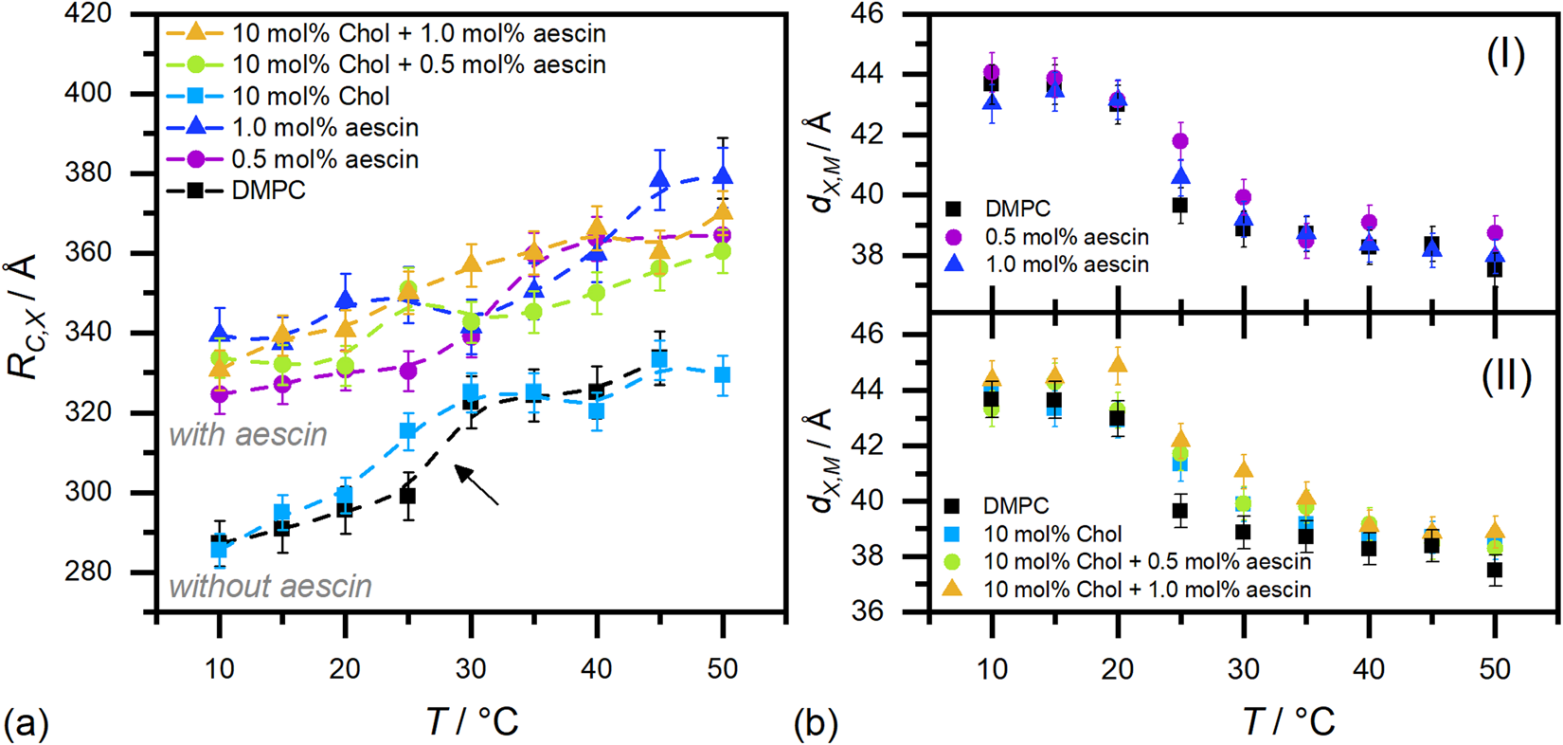
CMS fit results from the analysis of the SAXS curves in Fig. 6 and Supplementary Fig. S4. (**a**) Core radii *R*_*C*,*X*_ and (**b**) membrane thickness *d*_*X*,*M*_. Both parameters were obtained through analysis of the SAXS curves by the CMS model. The dashed lines in panel (a) shall improve readability of the figure. The poor data statistics of the SAXS curves in Fig. 6 and Supplementary Fig. S4 may have caused large error bars for the *R*_*C*,*X*_ values above 40 °C.

The *core multi shell* (CMS) model, described in Supplementary Fig. S3, was used to analyse the form factors of the SUVs^43–45^. This model is based on a core-shell structure with three shells to properly describe the complex X-ray scattering length density (XSLD) profile of a phospholipid bilayer seen by X-rays. Hereby, the inner and the outer shells stand for the headgroups (of DMPC and aescin) whereas the middle shell represents the hydrophobic portion taking into account two units of hydrocarbon tails of DMPC, the triterpenic backbone of aescin, and the full cholesterol molecule. The XSLD-values XSLD(head) and XSLD(tail) were calculated by taking the fraction of each compound in mol% into account. Values for pure DMPC are based on temperature-dependent measurements of the density by Nagle *et al*.^46^ Supplementary Table S1 summarises the parameters necessary for the XSLD calculation. Because thickness and XSLD parameters depend on each other, XSLD(head) and the headgroup thickness *d*_*h*_ were fixed. *d*_*h*_ was adjusted by fitting and best results were found for *d*_*h*_ = 7 Å. However, it must be noted that *d*_*h*_ takes the headgroups of DMPC and aescin into account. It lies in the range of reported values used in X-ray based analyses of ‘pure’ DMPC bilayers^32,47,48^. Moreover, the value used is slightly smaller than the typical one of 9 Å used in neutron-based analyses^49,50^. This seems reasonable when comparing contrasts of both methods (full bilayer in SANS vs. head-head thickness in SAXS). Additionally, XSLD(core) and XSLD(solvent) were adjusted starting from theoretical and temperature-corrected values for light water (see Supplementary Table S2). The inner core radius *R*_*C*,*X*_ and the hydrophobic thickness ${d}_{tail}={d}_{X,M}-2{d}_{h}$ were determined through fitting. Here, *d*_*X*,*M*_ is the total membrane thickness obtained by X-rays. *R*_*C*,*X*_ and *d*_*tail*_ were analysed with polydispersity (*σ*). For the former one a *Schulz* distribution and for the latter one a *Gaussian* distribution was applied. The Schulz distribution was used because the upper size limit of sample radii is artificially restricted by the extrusion procedure. The Gaussian distribution used for *d*_*tail*_ accounts for the presumed statistical variation of the hydrophobic membrane thickness and its (time- and space-) averaged fluctuations. The fit parameters are summarised in Supplementary Table S3. *σ* (*d*_*tail*_)-values did not change significantly and remained around 0–0.1. A scaling parameter and a background level were determined through fitting. The CMS fits are illustrated by solid lines in the corresponding figures. As can be seen, they are in good agreement with the experimental data. This confirms the vesicular shape of the samples. The characteristic shape of spherical objects is visible at low and the one of a bilayer structure at high *q*). The upturn at low–*q* indicates the onset of aggregation or deformation of SUVs at these aescin contents^51^. This is especially visible at the highest aescin concentration studied. Aggregates are assumed to be stabilised by attractive interactions between saponin molecules in adjacent bilayers. The fit results *R*_*C*,*X*_ and *d*_*X*,*M*_ from the CMS analyses are given in Fig. 7(a,b), respectively.

Samples with aescin exhibit higher *R*_*C*,*X*_ values than those without the saponin, while cholesterol has no evident effect. When aescin is present, its amount has only a marginal effect if any. The typical temperature-induced vesicle swelling is generally maintained for all systems (see also evolution fo *d*_*X*,*M*_). The sharp volumetric change around DMPC’s *T*_*m*_ is only seen for DMPC and SUVs with 0.5 mol% aescin. Higher *R*_*C*,*X*_ indicate either successful incorporation of aescin and/or probable deformation of SUVs leading to higher radii observed. Also, deformation of SUVs can be regarded as a result of successful incorporation of the saponin. In all cases, the mean radius is slightly larger than expected from an extrusion with 500 Å pores. Addition of cholesterol leads to the typical strong broadening around T_m_ (see arrow in panel (a)) indicating the disturbance of the well-ordered acyl chain packing by the stiff steroid^19^. This behaviour is maintained for systems with additional aescin. Since both effects (broadening of phase transition and higher *R*_*C*,*X*_) are present for the ternary systems we deduce successful incorporation of both compounds. Moreover, cholesterol was found to also smear the temperature-induced transition of the bilayer thinning due to volumetric changes of the hydrocarbon chains (Fig. 7(b,II)). This is in agreement with *R*_*C*,*X*_ results in panel (a). Also here, additional aescin conserves the smoother evolution of *d*_*X*,*M*_ with temperature in the cholesterol-containing systems (panel (II)). Cholesterol-free systems in panel (I) do show a sharper volumetric transition under similar conditions. These results are also in good agreement with the ones obtained from WAXS and DSC.

**Figure 8:**
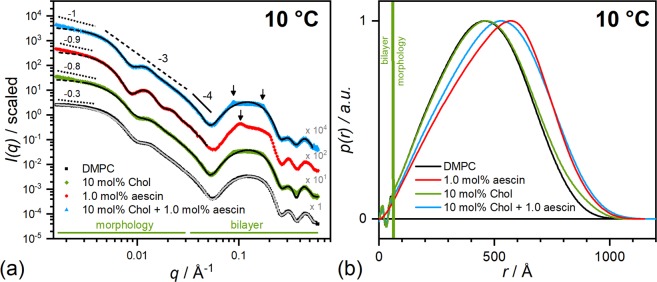
Model-independent SAXS results for samples with 1 mol% aescin at 10 °C. (**a**) Scattering curves and fits from the IFT analysis (solid lines). The data for DMPC is on absolute scale (cm^−1^). The other ones are scaled by the multiples in grey. The meaning of the slopes is explained in the main text (−3 and −4 apply for all curves). The *p*(*r*) functions calculated are shown in panel (b). Normalization from 0–1 guarantees better comparability.

To get a closer look onto the effect of cholesterol, model-independent analyses were performed on exemplary SAXS curves measured at 10 °C (Fig. 8). At this temperature the data has good statistics and most prominent impact of the saponin is expected.

The slopes of the SAXS curves in panel (a) of Fig. 8 were analysed and the scattering exponents *m* assigned to different kinds of structures (*I*(*q*) ~ *q*^−*m*^). Values for *m* = 1, 2, 3, and 4 denote cylinders, lamellae (disks), ~3D fractal objects, and sharp interfaces (Porod’s law), respectively^52–57^. Porods regime is given over $[1/{D}_{{\min }};\infty [$ where *D*_*min*_ is the shortest length^52^. In this *q*-range, the main contribution to the scattering intensity stems from interfaces between aggregates and decreases linearly with $m=4$:(${q}^{4}\cdot I(q)=2\pi {\rm{\Delta }}{\rho }^{2}\cdot {S}_{T}$). Here, ${\rm{\Delta }}{\rho }^{2}$ is the contrast resulting from the XSLD difference (or NSLD difference for SANS) and *S*_*T*_ the surface area of the aggregates per unit volume. Values for *m* of 3 and 4 are obtained for all samples. The first one arises from scattering of the bilayer of the SUV, the latter one from surface scattering, according to Porod’s law^52^. At lower *q*-values different slopes are obtained for the cholesterol and aescin-containing systems compared to pure DMPC SUVs where a plateau is reached. An increasing slope at very low *q* may indicate either scattering from the presence of very large SUVs (size distribution skewed towards higher radii) and/or aggregation of SUVs^57^. In the present system both components inserted are known to phase separate when embedded in phospholipid bilayers^19,41^. This leads to an inhomogeneous membrane and amongst others to elevated slopes at low-*q* in the scattering curves. This kind of structure formation was shown in former work for aescin-phospholipid mixtures^33^ but also for pure cholesterol-phospholipid systems by other groups^41^. Additionally to this effect, aggregation of vesicles is also visible in the bilayer structure of the scattering curve around 0.1 Å^−1^ in the form of appearing peak structures. A second peak at ${d}_{2}=2{d}_{1}$ (see Eqn. 1) denotes lamellar stacking. Whereas the cholesterol containing systems exhibit a similar position of the Bragg peaks with a correlation length of ≈72 Å, the cholesterol-free system containing aescin exhibits a rather disordered structure and has a shifted maximum position to smaller repeating distances (*D*_*X*,*RD*_ ≈ 62.8 nm). Smaller *D*_*X*,*RD*_ values indicate more compact packing of sheets and vice versa.

Additionally, SAXS curves were analysed by the standard IFT analysis (solid lines in the same figure) by using the GIFT software from O. Glatter^58^. The fitting procedure is described elsewhere^27^. The resulting pair distance distribution functions *p*(*r*) are shown in Fig. 8(b). The sample with 1 mol% aescin (*red*) was only analysed in the low-*q* part because of presence of peak structures in the high–*q* part. Stable solutions could not be found when the high-*q* part was included. The comparison of the low-*q* parts of the SAXS curves visualizes that additional cholesterol changes the average morphology of the structure induced by aescin. Whereas the sample without the steroid (red curve) shows distinct form factor oscillations, the one with additional cholesterol (blue) resembles more to the saponin-free ones (black and green). Strong form factor oscillations indicate low polydispersity of the sample. This effect is recognized in the *p*(*r*) functions. The maximum of the red curve representing the samples with aescin but without the steroid is inclined towards higher values of *r*. Hence, it corresponds to a rather ‘stiff’ hollow sphere compared to the other curves. Addition of cholesterol reverses this effect by reducing the degree of inclination and therewith confirming saponin-steroid complexation. This analysis yields furthermore a model-independent radius of gyration $({R}_{G}^{2}=\tfrac{\int \,p(r){r}^{2}{\rm{d}}r}{2\,\int \,p(r){\rm{d}}r})$ through integration of the *p*(*r*) function in real space^59,60^. *R*_*G*_-values for DMPC SUVs and SUVs with cholesterol are 335 Å and 354 Å, respectively. The ones with aescin exhibit larger values of 372 Å without and 385 Å with the steroid. These findings are in concordance with *R*_*C*,*X*_ values and with literature^61^.

### Small-angle neutron scattering (SANS)

**Figure 9:**
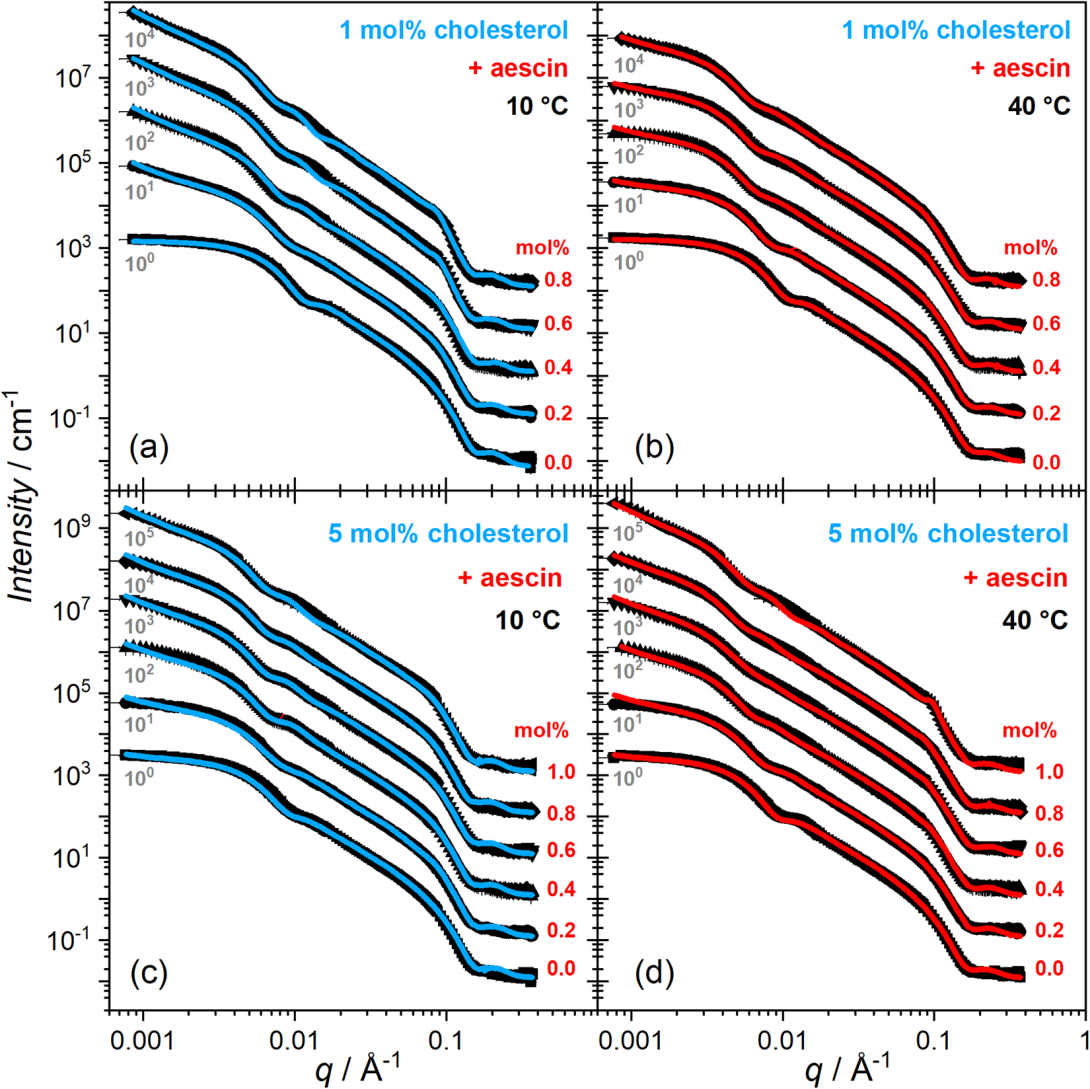
SANS curves of samples with (**a**,**b**) 1 mol% and (**c**,**d**) 5 mol% cholesterol with aescin contents between 0–1 mol% at 10 °C (blue curves) and 40 °C (red curves). Solid lines are model fits (vesicle + LS) by Eqn. 6. Red numbers denote the aescin content in mol%. SANS curves are scaled by the grey numbers.

SANS experiments were performed in order to elucidate the concentration-dependent aggregation and shape deformation of SUVs induced by low amounts of aescin (0–1 mol%) and cholesterol (1 and 5 mol%). SANS curves for samples containing 1 mol% cholesterol are shown in Fig. 9(a,b) and those with 5 mol% in panels (c) and (d). Temperatures investigated are 10 °C (blue fits) and 40 °C (red fits).

At these cholesterol and aescin contents two or in some cases three main structural effects were found to contribute to the measured intensity. The form factor which underlies all curves results from the vesicular shape of the extruded samples so that a form factor model for unilamellar vesicles is employed. The second effect results from vesicle-vesicle interactions. They alter the scattering intensity at very low *q*, lead to formation of a lamellar structure peak around 0.1 Å^−1^ and induce a very straight and linear scattering at intermediate *q* (0.01–0.06 Å^−1^). Elevated scattering at very low *q* (<0.004 Å^−1^) may result from shape deformation of SUVs through elongation of SUV structures and/or attractive interactions between them (e.g. by aggregation). The scattering exponent at low–*q* approaches a value of $m\approx 1.3$ for highest aescin content indicating scattering from rod-shaped or prolate elipsoidal particles. At intermediate *q* a scattering exponent of $m\approx 2$ is found, which is in concordance with SAXS data. This value for *m* denotes scattering from extended 2D sheets such as bilayers^52,54–56^. The last feature, i.e. peak formation around 0.1 Å^−1^ results from aggregation or stacking of bilayers^51^. As shown in a former study amongst others by cryogenic transmission electron microscopy (cryo-TEM)^33^, aescin has the tendency to form straight edges in unilamellar bilayered structures. Moreover, whereas SUV structures were found for samples <2 mol%, major structural reorganization was identified above 5–6 mol% of the saponin in the system. Nevertheless, similar features were not observed in SANS cuves for cholesterol free systems and similar aescin contents^27^. However, in this case it is assumed that the main phenomenon describing the structural scenario is aggregation between SUVs stabilised by inter-vesicular attractive saponin-saponin interactions. In this picture, bilayers of two SUVs approach and form a stack of two units^33^. Additionally, we assume modification of membrane properties by the incorporated saponin-steroid complexes. Finally, a summation of two models, the *vesicle*^45^ and the *paracrystal lamellar stack*^62^ (LS) model, was used to describe the scattering data. The combination of these models covers on one hand the vesicular character of the samples and on the other hand introduces additional interactions through multilamellarity which was identified in the high-*q* part. Both models are implemented in SASView^63^ and were combined and used for fitting. A schematic describing the models is shown in Supplementary Fig. S3.

The scattering intensity describing the unilamellar vesicle is given by Eqn. 3:

3 $$\begin{array}{rcl}I{(q)}_{ves} & = & P{(q)}_{ves}\\  & = & \frac{C}{{V}_{shell}}\,[\frac{3{V}_{core}({\rho }_{solvent}-{\rho }_{shell}){J}_{1}(q{R}_{core})}{q{R}_{core}}\\  &  & {+\frac{3{V}_{tot}({\rho }_{shell}-{\rho }_{solvent}){J}_{1}(q{R}_{tot})}{q{R}_{tot}}]}^{2}+background.\end{array}$$

Here, $P{(q)}_{ves}$ is the form factor of a SUV, *C* the adjustable scale factor, *V*_*shell*_ the volume of the shell, *V*_*core*_ the volume of the core, *V*_*tot*_ the total volume, ${R}_{core}={R}_{C,N}$ the radius of the core, *R*_*tot*_ the outer radius of the shell ${\rho }_{solvent}$ the neutron scattering length density (NSLD) of the core and the solvent, ${\rho }_{shell}$ the NSLD of the shell, and ${f}_{1}=(\sin \,x-x\,\cos \,x)/{x}^{2})$ the first-order spherical Bessel function. Hence, the vesicle is defined by its shell thickness *t* and its core radius *R*_*C*,*N*_.

The scattering intensity from the LS model used is calculated by Eqn. 4.

4 $$I{(q)}_{LS}=2\pi {({\rm{\Delta }}\rho )}^{2}{{\rm{\Gamma }}}_{m}\frac{{P}_{bil}(q)}{{q}^{2}}{Z}_{N}(q)\,{\rm{with}}\,{P}_{bil}={(\frac{\sin (qt/2)}{qt/2})}^{2}$$

This model uses a scale factor instead of the mass per area of the bilayer (${{\rm{\Gamma }}}_{m}$). Here, the scale factor is the volume faction of the material in the bilayer, not the total excluded volume of the paracrystal. The expression of *Z*_*n*_(*q*) in Eqn. 5 describes the interference effects for aggregates consisting of more than one bilayer.

5 $${Z}_{N}(q)=\frac{1-{w}^{2}}{1+{w}^{2}-2w\,\cos (q\langle D\rangle )}+{x}_{N}{S}_{N}+(1-{x}_{N}){S}_{N+1}$$

In this expression ${S}_{N}(q)={a}_{N}/N{[1+{w}^{2}-2w\cos (q\langle D\rangle )]}^{2}$ with ${a}_{N}=4{w}^{2}$ − $2({w}^{3}+w)\,\cos (q\langle D\rangle )$ − $4{w}^{N+2}\,\cos (Nq\langle D\rangle )$ + $2{w}^{N+3}\,\cos \,[(N-1)q\langle D\rangle ]$ + $2{w}^{N+1}\,\cos \,[(N+1)q\langle D\rangle ]$. The non-integer number of bilayer stacks *N*_*L*_ is calculated as linear combination of the lower and higher values ${N}_{L}={x}_{N}N+(1-{x}_{N})\,(N+1)$.

The final expression used for describing the SANS data is a summation of $I{(q)}_{ves}$ and $I{(q)}_{LS}$ (Eqn. 6) with *N* as adjustable scale factor and *a* and *b* denoting fractions of the vesicle and LS model, respectively.

6 $$I(q)=N\cdot [a\cdot I{(q)}_{ves}+b\cdot I{(q)}_{LS}]+background$$

The solid lines in Fig. 9 correspond to model functions obtained by Eqn. 6. The numerical results are summarised in Table 1.

**Table 1:** Results of the SANS data analysis.

*x*(*aescin*) mol%	*T*/°C	1 mol% cholesterol						5 mol% cholesterol					
		*N*_*L*_	NSLD/10^−6^ Å^−2^	*R*_*C*,*N*_/Å	*σ*(*R*_*c*_)/%	*a*	*b*	*N*_*L*_	NSLD/10^−6^ Å^−2^	*R*_*C*,*N*_/Å	*σ* (*R*_*c*_)/%	*a*	*b*
0.0	10	1	0.296	186 ± 0.3	38	1.00	0	1	0.293	225 ± 3	45	0.89	0.09
0.2	10	4	0.295	264 ± 0.3	30	0.63	0.38	3	0.277	295 ± 3	34	0.73	0.27
0.4	10	6	0.301	299 ± 2	30	0.43	0.57	4	0.298	356 ± 3	30	0.44	0.56
0.6	10	6	0.303	330 ± 2	27	0.28	0.72	6	0.300	386 ± 3	29	0.40	0.60
0.8	10	6	0.308	370 ± 2	22	0.20	0.80	6	0.302	360 ± 3	32	0.40	0.60
1.0	10	x	x	x	x	x	x	6	0.304	385 ± 3	28	0.29	0.71
0.0	40	1	0.277	208 ± 0.2	38	1.00	0	1	0.275	270 ± 3	28	0.91	0.09
0.2	40	3	0.280	260 ± 2	40	0.91	0.09	3	0.277	318 ± 2	30	0.67	0.33
0.4	40	4	0.282	305 ± 2	45	0.80	0.20	7	0.280	350 ± 3	40	0.65	0.36
0.6	40	4	0.284	320 ± 2	41	0.8	0.20	7	0.282	400 ± 3	35	0.50	0.50
0.8	40	4	0.287	334 ± 2	42	0.63	0.37	7	0.228	423 ± 3	37	0.44	0.56
1.0	40	x	x	x	x	x	x	8	0.287	445 ± 3	27	0.29	0.71

The parameters are the aescin content *x*(*aescin*), number of bilayers *N*_*L*_ rounded to whole numbers, the neutron scattering length density (NSLD), the core radius *R*_*C*,*N*_ and the fractions *a* and *b* (with a + b = 1), according to Eqn. 6.

The neutron scattering length densities (NSLDs) of the solvent were temperature corrected (${{\rm{NSLD}}}_{{{\rm{D}}}_{{\rm{2}}}{\rm{O}}}$(10 °C) = 6.37 · 10^−6^ Å^−2^ and ${{\rm{NSLD}}}_{{{\rm{D}}}_{{\rm{2}}}{\rm{O}}}$(40 °C) = 6.34 · 10^−6^ Å^−2^). The others were calculated with respect to the cholesterol and aescin content based on the temperature-independent NSLDs of both compounds: NSLD_Chol_ = 0.22 · 10^−6^ Å^−2^ and NSLD_aescin_ = 1.42 · 10^−6^ Å^−2^ ^33^. The NSLD of DMPC was also corrected on temperature and was calculated from the molar volumes at different temperatures as derived by Nagle *et al*.^46^. The values used are NSLD_DMPC_(10 °C) = 0.297 · 10^−6^ Å^−2^ and NSLD_DMPC_(40 °C) = 0.278 · 10^−6^ Å^−2^. Some of the parameters did not vary significantly for the samples or were kept constant and are therefore not mentioned explicitly in Table 1. Those parameters are the *scale* factor (≈0.019), the background (0.15), the bilayer spacing *D* (≈72 ± 2 Å), the relative standard deviation of the Gaussian layer distance distribution *σ*_*D*_/$\langle D\rangle$ (0.2 ± 0.1), and the bilayer thickness *t* (*t*(10 °C) = 40 ± 1 Å and *t*(40 °C) = 35 ± 1 Å).

The comparison of the model approximations with the scattering data in Fig. 9 shows that most of the curves follow the data with a reasonably good agreement. Hence, this model seems to represent both, the vesicular and the multilayered character of the samples, even though some of the samples show deviations at very low *q* and around 0.01 Å^−1^. It was found that the scattering curves cannot be sufficiently described when only assuming a stacking of two units. This is most likely due to other underlaying effects which have significant influence on the scattering intensity or possible (but not expected) formation of mulilayered vesicles, as indicated by the SANS data. On the one hand deformation and therewith elongation of the vesicles (shifting of the slope at low *q* towards −1) occurs during the aggregation. On the other hand, scattering from an inhomogeneous bilayer or formation of larger vesicles may increase the scattering intensity arbitrarily at low *q*^57^. Because of these many different contributions which cannot be well separated from each other, the fraction *b* ($a+b=1$) of the LS model will be used to approximate evolution of the amount or strength of aggregated SUVs in the samples under different conditions. Nevertheless, further analysis of the parameters obtained (see Table 1) shows that there are trends in dependence of aescin and cholesterol content. First, the core radius *R*_*C*,*N*_ increases with aescin content and temperature and is in general higher for samples with the higher cholesterol content and as expected at higher temperature due to the temperature-induced vesicle swelling^25^. This is conform with results obtained from SAXS. The parameters characterizing the amount of inter-vesicular interactions are the number of bilayers *N*_*L*_ and the fraction *b*. These two parameters are found to influence the shape of the model function mainly at very low *q* and around the bilayer correlation peak at 0.1 Å^−1^. Because the scattering intensity especially at very low *q* is influenced also by other effects (aggregation and homogeneity of the bilayer^57^) and the structural picture on the molecular level is not fully resolved, these two parameters should be compared in relative change rather than considered as absolute values. Here both, the values of *N*_*L*_ and *b* increase with increasing aescin content, denoting that the inter-vesicular interactions increase upon incorporation of more saponin. Also these interactions have more influence at lower temperature and higher cholesterol content. These results indicate that (most presumably) the number and size of aescin-cholesterol complexes lead to more and more intense interactions between adjacent vesicles and formation of more aggregated structures.

## Discussion and Conclusion

This work investigates structual parameters of DMPC model membranes in SUVs modified by the presence of aescin-cholesterol complexes. Wojciechowski *et al*.^64^ studied the effect of different saponins (except of aescin) in the presence of cholesterol on egg lecithin bilayers of different shapes. The study deals with bilayers extruded through 200 nm pores (LUVs), giant unilamellar vesicles (GUVs), and supported bilayers (SLBs). While they found that cholesterol significantly enhances the effect of surface active saponins, the results for all kinds of bilayers were consistent. However, the most pronounced effects were found for the high curvature lipid bilayers and haemolytic saponins. In contrast to the results for GUVs and supported bilayers, SUVs show saponin induced changes of shape and enhanced tendency to agglomeration. Therefore, we think that in our study the experimental results obtained for the SUVs produced by filtration through 50 nm pores will be qualitatively the same for other curvatures. While the general finding, i.e. the formation of aescin-cholesterol complexes, is expected to occur for all forms of model membranes, the bilayer curvature of the SUVs, which is higher than for LUVs and especially for GUVs, shall have an influence on the final shape of the vesicles and presumably the strength of their interaction.

In this work, the concentration range of compounds added ranged from 0–6 mol% aescin and 0–10 mol% cholesterol. At this cholesterol content the gel and fluid phase of the DMPC are still accessible. The structural analyses were only performed for samples with aescin contents up to 1 mol% to ensure vesicular shape of the model membrane. Aescin at higher contents leads to strong modification of sample appearance, as elaborated in previous works^25,26^. At these cholesterol contents, formation of ISCOMs is not expected.

DSC confirmed complex formation between aescin and cholesterol and outlined that this process depends on aescin and cholesterol in a concentration-dependent manner. These complexes alter the mostly visible aescin-induced phase separation and the shape of conserved SUVs at aescin contents <1 mol%. Systems with SUV structures (≤1 mol%) were investigated using WAXS, SAXS, and SANS in a quantitative and qualitative manner. These analyses provide insight into aescin-cholesterol interactions before major structural reorganization occurs. On the Å-length-scale, WAXS confirmed aescin-cholesterol complexation and indicated interaction on molecular scale. These experiments showed that the presence of cholesterol increases the overall acyl chain correlation distances and increases *A*_*L*_ in the gel phase. Addition of further aescin leads to similar relative changes in the cholesterol-containing and steroid-free system. Larger length scales were covered by SAXS and SANS. Whereas SAXS experiments focussed on the temperature-dependency of the systems studied, SANS was exploited to follow structural changes depending on concentration. SAXS showed that the cholesterol binds to the aescin and alters the shape of structures. Nevertheless, the temperature-dependence of the overall structures was influenced by both compounds added. The aspect of aggregation/deformation and appearance of multilamellar correlations, seen in the bilayer structure in SAXS, was also analysed for different aescin and cholesterol contents by SANS. These experiments reveal that the multi layered character (and proceeding aggregation and deformation) of samples increases with increasing aescin and cholesterol content. Finally, this work has shown, that aescin-cholesterol-complexes are formed in a phosphocholine membrane. This complex formation might induce pore formation inside the bilayer and thereby significantly modify the membrane permeability. However, this effect cannot be directly derived from this work and should be addressed in a separate study.

## Methods

### Materials

1,2-dimyristoyl-*sn*-glycero-3-phosphocholine (DMPC) was purchased from Lipoid GmbH (>99%, Ludwigshafen, Germany). The pure saponin aescin (≥95%, CAS number 6805-41-0), cholesterol (≥95%), and chloroform were obtained from Sigma Aldrich (Munich, Germany). Samples for DSC, SAXS and WAXS were prepared with deionized, purified water (Sartorius arium VF pro, Göttingen, Germany). SANS samples were prepared in deuterium oxide (>99%, Carl Roth GmbH + Co. KG, Karlsruhe, Germany). Since aescin dissolves poorly in water and decreases the pH significantly, a 50 mM phosphate buffer (pH 7.4/pD 7.8) was used to guarantee a constant pH and solubility in aqueous solution. DMPC was used at a mass concentration of 15 g · L^−1^. The aescin concentration used (in mol%) is calculated by taking into account all components (DMPC and cholesterol) in solution (DMPC, aescin, and cholesterol): $x(aescin)={n}_{aescin}/({n}_{aescin}+{n}_{DMPC}+{n}_{cholesterol})$. The cholesterol concentration is defined in dependence of the amount of DMPC: $x(cholesterol)={n}_{cholesterol}/({n}_{DMPC}+{n}_{cholesterol})$.

### Sample preparation

The samples were prepared by the lipid film hydration method. For this, DMPC powder was dissolved in chloroform which was subsequently evaporated slowly by using a rotary evaporator. For complete chloroform evaporation, the sample was dried in an oven over night at 60 °C. The thin lipid film obtained was hydrated with an aescin solution in aqueous phosphate buffer solution. This procedure yields multilamellar vesicles (MLVs) which were enlarged with 5 freeze-thaw cycles. Small unilamellar vesicles (SUVs) were finally produced by extrusion (21×) at around 40 °C. The extruder (Avanti Polar Lipids Inc., Alabama, USA) used was equipped with a polycarbonate membrane (Whatman, Avanti Polar Lipids, Alabama, USA) with a pore diameter of 500 Å.

### Differential scanning calorimetry (DSC)

The thermotropic phase behaviour of the phospholipid bilayer was investigated by DSC. The experiments were carried out on a differential scanning heat-flow calorimeter (Q100, TA Instruments, New Castle, USA, referenced to air due to calibration of the instrument (see^65^)). Approximately 20 mg of the samples were hermetically sealed into aluminium pans (TA Instruments) and placed into the calorimeter. For thermalisation prior to the experiment, the sample was heated to 40 °C, hold at this temperature for 8 min, and subsequently cooled down with a constant cooling ramp of 7 °C/min to 7 °C. Again, the sample was thermalised at this temperature for 8 min before recording the thermograms from 7 °C to 40 °C with a scan rate of 0.50 °C · min^−1^. In this experiment, the heat flow is measured between the sample and the reference (air) in dependence of temperature. The specific heat capacity (${C}_{p,diff}$) is normalized to the sample mass.

### Small-angle and wide-angle scattering

Small-angle and wide-angle scattering techniques are widely used to determine structural and mechanical parameters of model membranes^27,49,59,66–68^. The complementary use of neutrons and X-rays is advantageous because both methods are sensitive to different contrasts. The kind of contrast determines which structural features become visible. Neutron scattering is sensitive to the neutron scattering length density (NSLD) difference, X-ray scattering is sensitive to the electron density (ED). Because of this, X-ray scattering is very suitable to study interfaces formed by amphiphiles, such as phospholipids^59,68^. Here, the highest ED is located around the phospholipid headgroups. On the other hand, neutrons benefit from the high NSLD difference between the hydrocarbon chains and the deuterated solvent so that the total bilayer thickness can be determined accurately.

The scattering intensity $I(q)=N\cdot {({\rm{\Delta }}\rho )}^{2}\cdot {V}^{2}\cdot P(q)\cdot S(q)$ is detected by the detector and depends on the scattering volume of the particle (*V*), the scattering length density (SLD) difference ${\rm{\Delta }}\rho ={\rho }_{{\rm{particle}}}-{\rho }_{{\rm{solvent}}}$, the form factor *P*(*q*), and the structure factor *S*(*q*). The scattering vector magnitude is $q=\frac{4\pi }{\lambda }\cdot \,\sin (\theta )$ where *θ* corresponds to half of the scattering angle 2*θ*.

#### Wide-angle X-ray scattering (WAXS)

Temperature-dependent (10–50 °C) WAXS experiment were performed for pure DMPC SUVs and SUVs with additional aescin (0.5 and 1 mol%) and cholesterol (0 and 10 mol%). The resolved *q*–range was 0.6–4 Å^−1^ at a sample to detector distance of 0.12 m. The cholesterol-free samples were measured 10 × 1 s and the steroid-containing ones 25 × 1 s with a CCD Rayonix LX-170HS detector and a wavelength of 1 Å. The first data treatment was done with the beamlines data reduction program package^69^. The samples were measured in a flow-through Kapton capillary (1 mm, Good-Fellow GmbH, Bad Nauheim, Germany) which is temperature-controlled in a Linkam stage (Linkam Scientific, Tadworth, UK). The sample scattering was normalized by sample thickness, the incident intensity, transmission, background, and acquisition time. Water was used for absolute intensity calibration.

#### Small-angle X-ray scattering (SAXS)

SAXS was performed for DMPC SUVs and SUVs with 1 mol% aescin. Supplementary cholesterol was varied between 0 and 10 mol%. The scattering curves were recorded simultaneously to WAXS at the ID02 beamline at the ESRF synchrotron in Grenoble, France^70^. The *q*-regime resolved is 1.5 · 10^−3^–0.6 Å^−1^.

#### Small-angle neutron scattering (SANS)

SANS experiments were realized at the D11^71^ instrument at the Institut Laue Langevin (ILL), Grenoble, France. The samples were filled into 2 mm quartz cuvettes (Hellma Analytics, Müllheim, Germany) and measured in an automated sample changer with 9 positions first at 10 °C and then at 40 °C. A *q*-range from 7.6 · 10^−4^ Å^−1^ to 0.4 Å^−1^ was covered using a neutron wavelength of 6 Å at sample-detector-distances of 2 m, 8 m, and 39 m. Additionally experiments with 13 Å and 39 m were performed. The wavelength resolution was Δ*λ*/*λ* = 10%. Initial treatment of the 2D data was done with the lamp software provided by the ILL. Absolute scale was obtained by using light water as a secondary standard. The ILL raw data have already obtained a DOI^72^.

## Supplementary information

Supplementary data
