# Supplementary material for: Aescin-Cholesterol Complexes in DMPC Model Membranes: A DSC and Temperature-Dependent Scattering Study
Source: Sci Rep. 2019 Apr 3;9:5542. doi: 10.1038/s41598-019-41865-z (PMC6447539; doi:10.1038/s41598-019-41865-z)
Supplement: Supplementary file 1 — Supplementary data [file 41598_2019_41865_MOESM1_ESM.pdf]

Electronic Supplementary Information

Aescin-Cholesterol Complexes in DMPC Model Membranes: A DSC and Temperature-Dependent Scattering Study

Ramsia Sreij,[a] Carina Dargel,[a] Ralf Schweins[b], Sylvain Prévost,[b,c] Rajeev Dattani,[c] and Thomas Hellweg,*[a]

*Email : thomas.hellweg@uni-bielefeld.de
*Phone : +49(0)521 1062055 Fax : +49(0)521 1062981

[a] Physical and Biophysical Chemistry, Bielefeld University, Universitätsstr. 25, 33615 Bielefeld, Germany.

[b] Institut Laue-Langevin, DS/LSS, 71 avenue des Martyrs, 38042 GRENOBLE Cedex 9, France

[c] ESRF-The European Synchrotron, 71, Avenue des Martyrs, 38043 Grenoble Cedex 9,France.

1. Wide-angle X-ray scattering (WAXS)

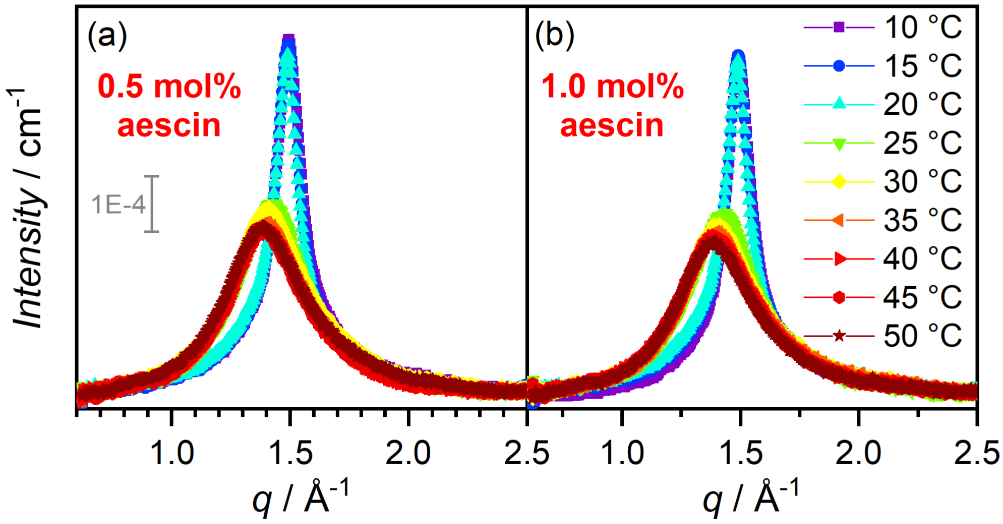

Figure S1 WAXS curves. Wide-angle X-ray scattering (WAXS) spectra at temperatures around the main phase transition temperature T_m of DMPC and aescin containing (a) 0.5 mol% aescin and (b) 1.0 mol% aescin. The scale bar applies for all panels. Please see electronic version for color encoding.

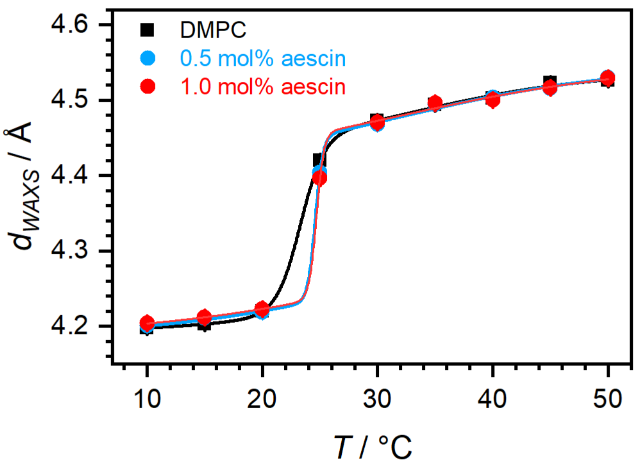

Figure S2: Acyl chain correlation distances d_{WAXS} calculated from peak maxima in Fig. S1 by Eqn. 1 in the main article. The figure shows samples without cholesterol. Solid lines are sigmoidal fits to the data.

2. Small-angle scattering (SAXS and SANS)

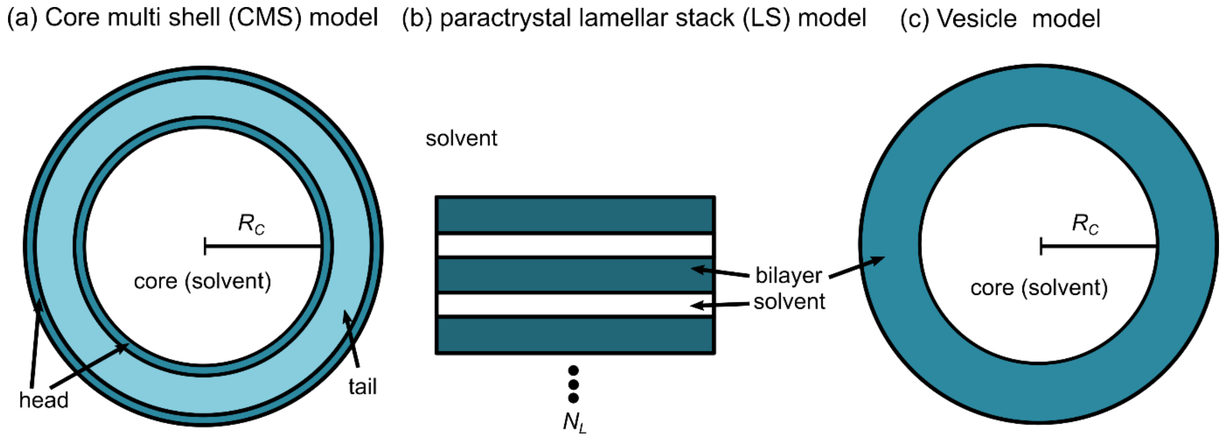

Figure S3: Schematics of models used for fitting. (a) *Core multi shell* (CMS) model. (b) *Vesicle* model (c) *Paracrystal lamellar stack* (LS) model. All models are implemented in SASView[1]. The CMS model was used for SAXS and the LS and Vesicle models for SANS analysis.

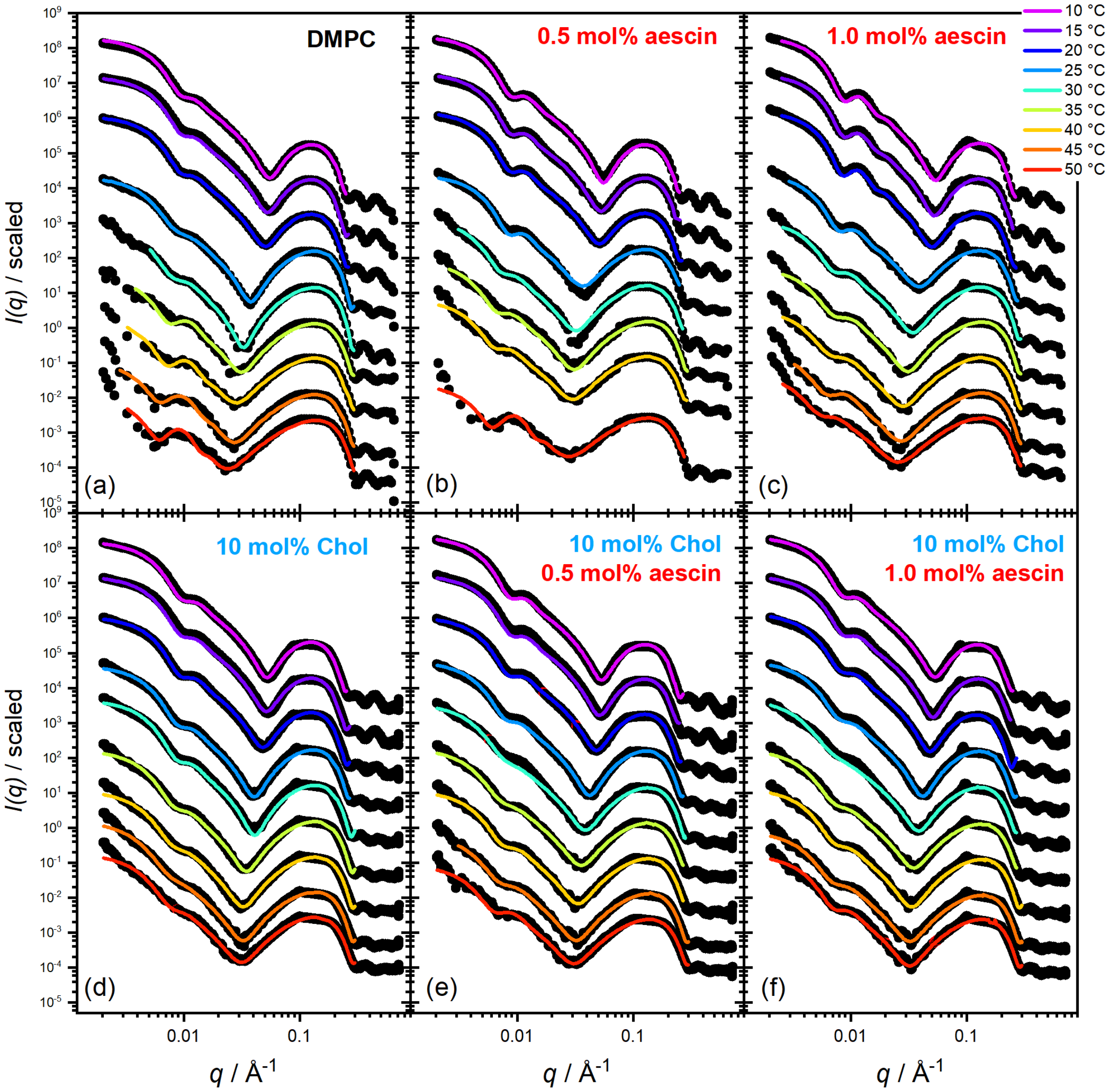

Figure S4: SAXS curves (a)-(c) without and (d)-(f) with 10 mol% cholesterol at temperatures between 10 °C and 50 °C, measured at ID02 beamline. Samples in panels (a) and (d) are without aescin while (b) and (e) contain 0.5 mol% aescin and (c) and (f) 1 mol% aescin. Solid lines correspond to the *core multi shell* (CMS) model implemented in SASView.[1] XSLDs used for fitting are summarised in Table S1. The scattering curves at 50 °C are on absolute scale (cm^{-1}). All other ones are scaled with respect to it (by factors of 10).

Table S1 Parameters used to calculate the XSLD for DMPC, aescin and cholesterol. The parameters are molecular formula, density (ρ), molar volume (V_M) and molar mass (M), the molecular volume (V) and the X-ray scattering length density (XSLD). Values for aescin were calculated with ChemSketch[2]. ρ (DMPC) are temperature-dependent. All other values are independent on temperature. Values for DMPC are based on experiments by Nagle et al.[3]. Bold values were directly taken from this study.

component	**Molecular formula**	ρ g/cm³	V_M cm³ / mol	M g/mol	V Å³	*XSLD* 10^{-6} Å²
Cholesterol	$C_{27}H_{46}O$	0.98	391.4	386.7	630[5]	9.32
Aescin	$C_{55}H_{86}O_{24}$	1.46	771.1	1131.3	1280	13.4
Aescin (head)	$C_{18}H_{30}O_{17}$	1.88	275.4	518.4	457	16.9
Aescin (tail)	$C_{37}H_{58}O_7$	1.16	527.6	614.8	876	10.8
DMPC	$C_{36}H_{72}NO_8P$	**1.08**	626.4	677.9	**1040**	10.2
DMPC(head)	$C_{10}H_{18}NO_8P$	1.50	207.2	311.2	**344**	13.5
DMPC(tail)	$C_{26}H_{54}$	-	419.3	366.8	696	-

Table S2: Calculated XSLDs for light water (H_2O) in dependence of temperature.

***T* /** °C	**10**	**15**	**20**	**25**	**30**	**35**	**40**	**45**	**50**
**XSLD /** 10^{-6} Å^2	9.47	9.46	9.46	9.44	9.43	9.42	9.40	9.38	9.36

Small-angle X-ray scattering – Fit parameters – Core-multi-shell fit (CMS)

Table S3: XSLDs used for fitting of the SAXS data with the CMS Model from SASView (V 4.1.2.)[1] The fit parameters XSLD(head) and d_h=7 Å were fixed for both shells prior to fitting. The parameters XSLD(solvent) and XSLD(core) were fitted starting from calculated values for light water (H_2O). XSLD(tail) was the final parameter to adjust due to the most uncertain XSLD value because of volumetric changes and temperature sensitivity. The structural parameters obtained from the fit ($R_{C,X}$ and d_M=d_h + d_{tail} + d_h) are plotted in Fig. 7 in the main manuscript.

**x(aescin)**	**X(Chol)**	***T***	**XSLD (head)**	**XSLD(tail) calc**	**XSLD(tail) fit**	**XSLD (solvent)**	**XSLD (core)**	**PDI**
mol%	mol%	°C	10^{-6} Å^{-2}	10^{-6} Å^{-2}	10^{-6} Å^{-2}	10^{-6} Å^{-2}	10^{-6} Å^{-2}	$R_{C,X}$
0	0	10	13.500	8.520	8.324	9.472	9.522	0.25
0	0	15	13.500	8.400	8.281	9.470	9.513	0.25
0	0	20	13.500	8.320	8.125	9.453	9.498	0.26
0	0	25	13.500	7.910	7.588	9.443	9.450	0.28
0	0	30	13.500	7.840	7.395	9.431	9.433	0.28
0	0	35	13.500	7.790	7.294	9.417	9.407	0.24
0	0	40	13.500	7.730	7.118	9.375	9.365	0.26
0	0	45	13.500	7.700	7.119	9.352	9.337	0.25
0	0	50	13.500	7.560	7.037	9.341	9.333	0.20
**x(aescin)**	**X(Chol)**	***T***	**XSLD (head)**	**XSLD(tail) calc**	**XSLD(tail) fit**	**XSLD (solvent)**	**XSLD (core)**	**PDI**
mol%	mol%	°C	10^{-6} Å^{-2}	10^{-6} Å^{-2}	10^{-6} Å^{-2}	10^{-6} Å^{-2}	10^{-6} Å^{-2}	$R_{C,X}$
0.5	0	10	13.517	8.531	8.375	9.470	9.506	0.22
0.5	0	15	13.517	8.412	8.186	9.445	9.482	0.21
0.5	0	20	13.517	8.332	8.137	9.448	9.476	0.22
0.5	0	25	13.517	7.924	7.747	9.474	9.476	0.23
0.5	0	30	13.517	7.855	7.594	9.419	9.423	0.27
0.5	0	35	13.517	7.805	7.282	9.396	9.391	0.27
0.5	0	40	13.517	7.745	7.258	9.398	9.394	0.27
0.5	0	45	13.517	not measured				
0.5	0	50	13.517	7.576	7.169	9.370	9.355	0.16
**x(aescin)**	**X(Chol)**	***T***	**XSLD (head)**	**XSLD(tail) calc**	**XSLD(tail) fit**	**XSLD (solvent)**	**XSLD (core)**	**PDI**
mol%	mol%	°C	10^{-6} Å^{-2}	10^{-6} Å^{-2}	10^{-6} Å^{-2}	10^{-6} Å^{-2}	10^{-6} Å^{-2}	$R_{C,X}$
1.0	0	10	13.534	8.534	8.221	9.456	9.494	0.18
1.0	0	15	13.534	8.424	8.227	9.452	9.485	0.19
1.0	0	20	13.534	8.345	8.145	9.453	9.487	0.17
1.0	0	25	13.534	7.939	7.612	9.442	9.445	0.22
1.0	0	30	13.534	7.870	7.396	9.429	9.428	0.30
1.0	0	35	13.534	7.820	7.296	9.414	9.408	0.25
1.0	0	40	13.534	7.761	7.189	9.395	9.390	0.28
1.0	0	45	13.534	7.731	7.123	9.369	9.362	0.24
1.0	0	50	13.534	7.592	7.007	9.347	9.339	0.26

Table S3 (continuation): XSLDs used for fitting of the SAXS data with the CMS Model from SASView (V 4.1.2.)[1] The fit parameters XSLD(head) and d_h=7 Å were fixed for both shells prior to fitting. The parameters XSLD(solvent) and XSLD(core) were fitted starting from calculated values for light water (H_2O). XSLD(tail) was the final parameter to adjust due to the most uncertain XSLD value because of volumetric changes and temperature sensitivity. The structural parameters obtained from the fit ($R_{C,X}$ and $d_M = d_h + d_{tail} + d_h$) are plotted in Fig. 7 in the main manuscript.

**x(aesc)**	**X(Chol)**	***T***	**XSLD (head)**	**XSLD(tail) calc**	**XSLD(tail) fit**	**XSLD (solvent)**	**XSLD (core)**	**PDI**
mol%	mol%	°C	10^{-6} Å^{-2}	10^{-6} Å^{-2}	10^{-6} Å^{-2}	10^{-6} Å^{-2}	10^{-6} Å^{-2}	$R_{C,X}$
0	10	10	13.500	8.600	8.295	9.475	9.506	0.25
0	10	15	13.500	8.492	8.219	9.466	9.505	0.25
0	10	20	13.500	8.420	8.092	9.463	9.494	0.26
0	10	25	13.500	8.051	7.818	9.466	9.486	0.25
0	10	30	13.500	7.988	7.660	9.434	9.451	0.25
0	10	35	13.500	7.943	7.449	9.424	9.433	0.28
0	10	40	13.500	7.889	7.373	9.423	9.428	0.30
0	10	45	13.500	7.862	7.305	9.382	9.389	0.35
0	10	50	13.500	7.736	7.235	9.363	9.366	0.30
**x(aescin)**	**X(Chol)**	***T***	**XSLD (head)**	**XSLD(tail) calc**	**XSLD(tail) fit**	**XSLD (solvent)**	**XSLD (core)**	**PDI**
mol%	mol%	°C	10^{-6} Å^{-2}	10^{-6} Å^{-2}	10^{-6} Å^{-2}	10^{-6} Å^{-2}	10^{-6} Å^{-2}	$R_{C,X}$
0.5	10	10	13.517	8.611	8.261	9.457	9.501	0.22
0.5	10	15	13.517	8.804	8.265	9.445	9.484	0.23
0.5	10	20	13.517	8.432	8.198	9.463	9.484	0.23
0.5	10	25	13.517	8.065	7.827	9.436	9.452	0.27
0.5	10	30	13.517	8.003	7.548	9.437	9.452	0.33
0.5	10	35	13.517	7.958	7.521	9.422	9.427	0.28
0.5	10	40	13.517	7.904	7.382	9.400	9.403	0.27
0.5	10	45	13.517	7.878	7.226	9.357	9.353	0.25
0.5	10	50	13.517	7.752	7.180	9.359	9.352	0.30
**x(aescin)**	**X(Chol)**	***T***	**XSLD (head)**	**XSLD(tail) calc**	**XSLD(tail) fit**	**XSLD (solvent)**	**XSLD (core)**	**PDI**
mol%	mol%	°C	10^{-6} Å^{-2}	10^{-6} Å^{-2}	10^{-6} Å^{-2}	10^{-6} Å^{-2}	10^{-6} Å^{-2}	$R_{C,X}$
1.0	10	10	13.534	8.623	8.364	9.478	9.516	0.23
1.0	10	15	13.534	8.516	8.315	9.479	9.512	0.23
1.0	10	20	13.534	8.445	8.250	9.477	9.504	0.25
1.0	10	25	13.534	8.080	7.861	9.440	9.458	0.27
1.0	10	30	13.534	8.018	7.692	9.436	9.450	0.38
1.0	10	35	13.534	7.973	7.520	9.422	9.427	0.30
1.0	10	40	13.534	7.920	7.382	9.401	9.406	0.27
1.0	10	45	13.534	7.893	7.289	9.378	9.377	0.29
1.0	10	50	13.534	7.768	7.264	9.360	9.361	0.25

References

[1] SASView, Freeware Version 4.1.2, http://www.sasview.org, 2017.

[2] ACD/ChemSketch, Freeware Version, Advanced Chemistry Development, Inc., Toronto, ON, Canada, www.acdlabs.com, 2015.

[3] Nagle, J. F. and Wilkinson, D. A., Lecithin bilayers. Density measurement and molecular interactions, Biophys. J., **23**(2), 159-175 (1978).

[4] Porod, G. Die Röntgenkleinwinkelstreuung von dichtgepackten kolloidalen Systemen – I. Teil- *Kolloid Zeitschrift*, **124**, 83-114 (1951).

[5] Greenwood, A. I., Tristam-Nagle, S. & Nagle, J. F. Partial molecular volumes of lipids and cholesterol, *Chemistry and physics of lipids*, **114**(1-2), 1-10 (2006)
